# Long-acting acid-sensitive ketal-linked dexamethasone microcrystals for treating experimental autoimmune uveitis

**DOI:** 10.1063/5.0118311

**Published:** 2022-10-26

**Authors:** Maoyu Cai, Zunkai Xu, Xueyan Zhou, Liangpin Li, Xia Hua, Shutao Guo, Xiaoyong Yuan

**Affiliations:** 1 Clinical College of Ophthalmology, Tianjin Medical University, Tianjin 300020, China; 2 Tianjin Key Laboratory of Ophthalmology and Visual Science, Tianjin Eye Institute, Tianjin Eye Hospital, Tianjin 300020, China; 3 Key Laboratory of Functional Polymer Materials of Ministry of Education, State Key Laboratory of Medicinal Chemical Biology and Institute of Polymer Chemistry, College of Chemistry, Nankai University, Tianjin 300071, China; 4 School of Medicine, Nankai University, Tianjin 300071, China; 5 Aier Eye Institute, Changsha 410015, China; 6 Tianjin Aier Eye Hospital, Tianjin 300190, China

## Abstract

Corticosteroids have for some time been used as first-line drugs for the topical treatment of noninfectious uveitis, but poor ocular bioavailability and the rapid clearance of eye drops necessitate frequent dosing, reducing patient compliance. In this study, we used an acid-sensitive stearoxyl-ketal-dexamethasone pro-drug microcrystals (SKD MCs), which is consistently safe and effective in the control of uveitis inflammation in rats. We used a rat model of experimental autoimmune uveitis (EAU) to evaluate the effects of SKD MCs in terms of clinical manifestations, molecular biology, pathological histology, and visual electrophysiology compared to dexamethasone sodium phosphate injection or phosphate-buffered saline. SKD MCs significantly reduced inflammation in EAU, improved the ability to suppress inflammatory cytokines and to protect retinal function, and significantly reduced retinal microglia activation, with no increase in intraocular pressure throughout the treatment. Our results indicate that the SKD MCs formulation holds promise as a new strategy for the treatment of noninfectious uveitis and potentially other ocular inflammatory diseases.

## I. INTRODUCTION

Uveitis is a serious potentially blinding ocular disease, which accounts for 5%–20% of all cases of blindness in the United States and Europe,^1,2^ occurs mostly in patients of working age (20–50 years),^2,3^ and is becoming an urgent problem due to its socioeconomic impact.^2^ Uveitis is classified etiologically as infectious and noninfectious, noninfectious uveitis (NIU) being idiopathic or associated with systemic autoimmune diseases, and the more common type in developed countries. Long-term control of NIU is needed to prevent the disease from damaging vision, but the effectiveness of existing treatment is limited by poor patient compliance over long periods.^4^

For human uveitis, both topical and systemic uses of steroids are common and effective therapies.^5^ While corticosteroids administered topically are effective in the treatment of uveitis, they have low bioavailability and require frequent administration, demanding high patient compliance. Severe intermediate or posterior uveitis may require periocular or intravitreal injections,^6^ further increasing treatment difficulties. For severe vision-threatening uveitis, especially when accompanied by cystoid macular edema, the standard treatment is systemic drug therapy with oral corticosteroids, which have a range of potential side effects.^7,8^ Therefore, a long-acting formulation is needed to improve the effectiveness of treatment by reducing the frequency of dosing and the side effects.

Intravitreal injections of corticosteroids are effective in the treatment of inflammation and are commonly used to control intraocular inflammation.^5^ In recent years, Ozurdex^®^, a bioerodible implant consisting of a mix of polylactic acid and polyglycolic acid polymers, has been commonly used to allow the sustained release of poorly water-soluble dexamethasone for up to six months.^9^ While intravitreal administration is accompanied by injection-related risks, subconjunctival drug delivery is considered safe and effective with many advantages compared to other routes of administration. It has been demonstrated that subconjunctival injections of dexamethasone sodium phosphate are effective in delivering the drug to the anterior and posterior segments of the eye.^10^ Many long-acting injectable formulations have been developed in recent years to extend drug retention time. Prodrugs are formed by combining a drug with hydrophobic promoieties, which reduce the solubility of native drugs, and show promise in the development of long-term extended release formulations.^12,13^

**FIG. 1.**
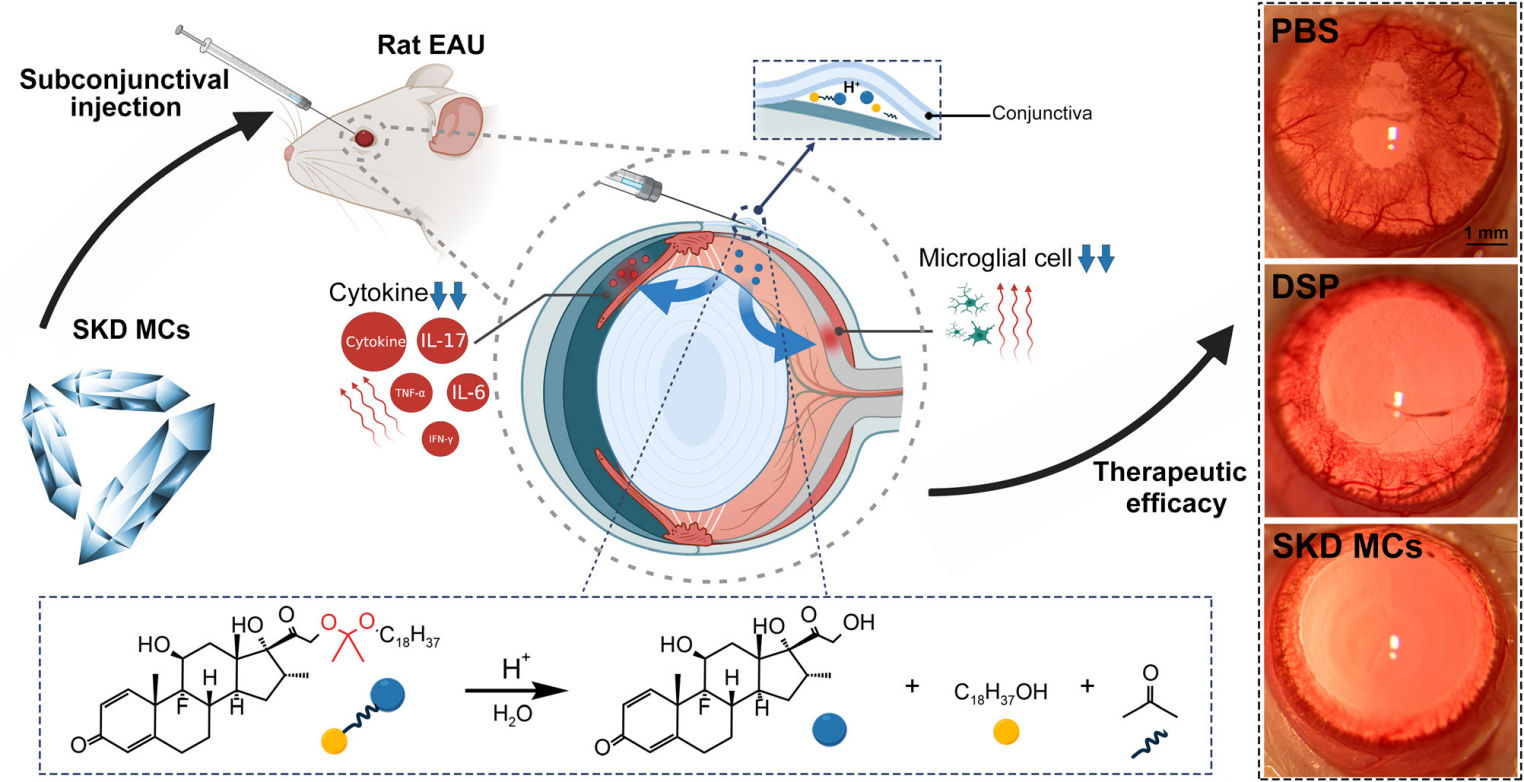
Schematic illustration of the study protocol. Stearoxyl-ketal-dexamethasone pro-drug microcrystals (SKD MCs) were used to treat experimental autoimmune uveitis (EAU) in rats via a single subconjunctival injection. This study demonstrated that by suppressing the expression of inflammatory cytokines and activation of retinal microglia, SKD MCs achieved a long-lasting effect, with sustained reduction of inflammation. The right-hand panel shows three slit lamp images of anterior eyes (with dilated pupils) of rats with EAU on different treatments at day 18 post-immunization. In comparison to dexamethasone sodium phosphate (DSP) and phosphate-buffered saline (PBS), SKD MCs significantly reduced the dilatation of iris vessels and pupillary adhesions in EAU rats at day 18 post-immunization. In the central panel, red arrows indicate our processing of EAU rats (injection of SKD MCs into the subconjunctiva, and the results in EAU rats after different treatments). In the lower panel, yellow circles indicate stearyl alcohol, blue circles indicate dexamethasone, and distorted connecting lines indicate ketal bonds.

It is well known that the pH at inflammatory sites is acidic.^14^ Acid-sensitive prodrugs can be hydrolyzed on demand at the site of inflammation and re-release the drug through an acid response when inflammation recurs. Here, we induced an experimental autoimmune uveitis (EAU) model in rats and subsequently evaluated drug efficacy using a single subconjunctival injection of Stearoxyl-ketal-dexamethasone pro-drug microcrystals (SKD MCs) (Fig. 1).

## II. RESULTS

### A. The efficacy of SKD MCs in an EAU rat model

**FIG. 2.**
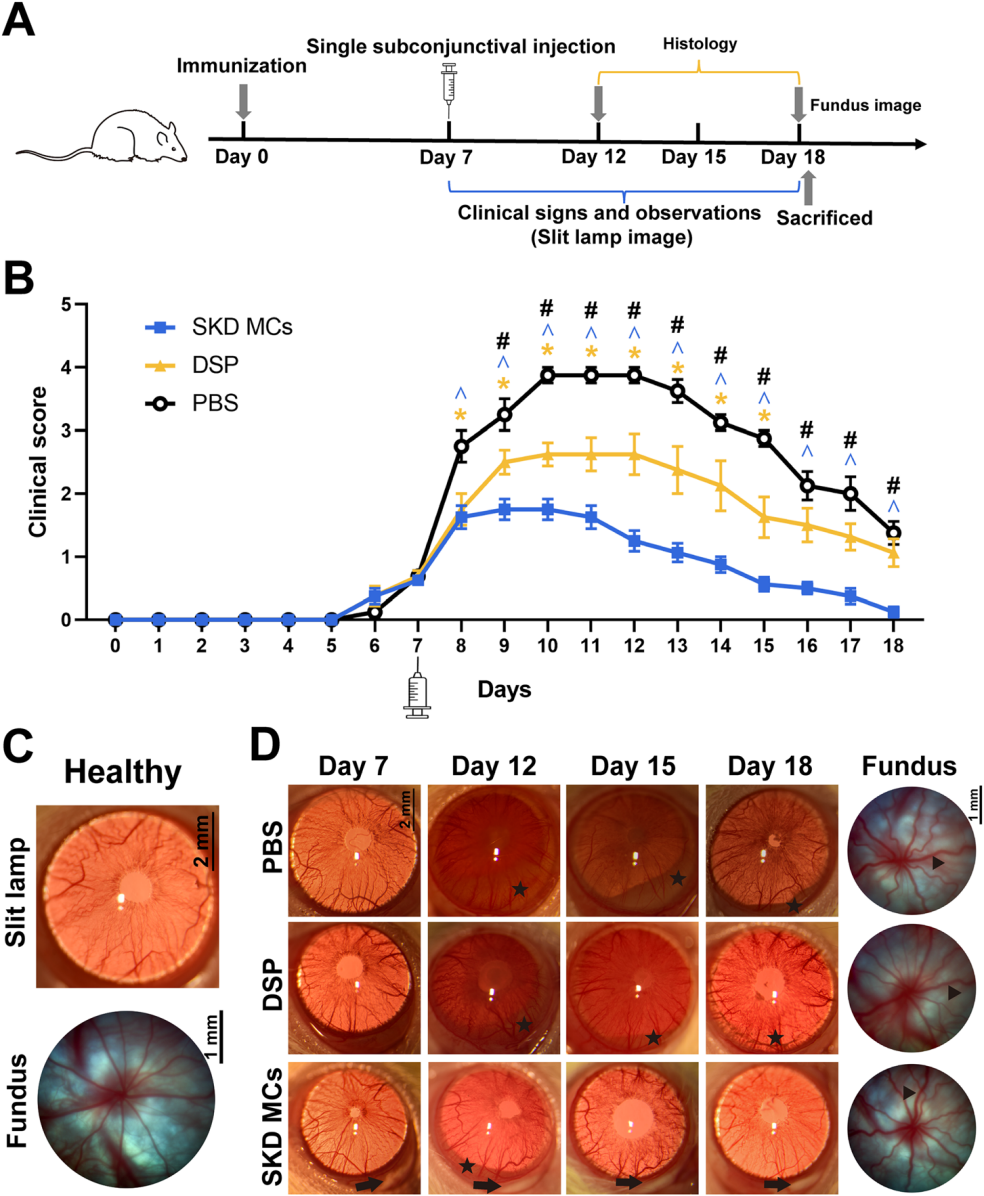
Therapeutic effect of stearoxyl-ketal-dexamethasone pro-drug microcrystal (SKD MCs) in an experimental anterior uveitis (EAU) rat model. (a) Schematic of EAU model establishment and treatment schedule. (b) Anterior segment disease score of rats in each group as a function of time. (c) Slit lamp anterior segment images and fundus images of healthy rats. (d) Slit lamp anterior segment images and fundus images of EAU rats under different treatments. Black stars indicate hypopyon, the black arrow indicates that the drug is continuously in place, and black triangles indicate tortuous dilatation of blood vessels in the fundus. *P <* *0.05 (PBS vs DSP); ∧*P *<* *0.05, (PBS vs SKD); #*P* <* *0.05 (SKD MCs vs DSP). Mean ± SEM, n = 8, one-way ANOVA test. PBS = phosphate-buffered saline; DSP = dexamethasone sodium phosphate.

We evaluated the advantage of SKD MCs by observing the effects of different drugs in a model of EAU in which all rats were divided into three groups and treated with different therapies [Fig. 2(a)]. In this model, the inflammatory response appeared on day 6 post-immunization, and on day 7 post-immunization, the rats were given a single subconjunctival injection of 40 *μ*l PBS (6.7 mM PBS containing 0.5% polysorbate 80), DSP (5 mg/ml), or SKD MCs (5 mg/ml eq. dexamethasone). The subsequent clinical scores gradually increased until they reached their maximum on days 10–12. From the day after subconjunctival injection (day 8 post-immunization), clinical scores were significantly higher in the PBS group than in the other two groups (*P *<* *0.05) [Fig. 2(b)]. From day 9 post-immunization until the end of the observation cycle (day 18 post-immunization), scores were significantly lower in the SKD MCs group than in the DSP group (*P *<* *0.05) [Fig. 2(b)].

In healthy rats, the iris vessels are clearly visible, and the fundus red light reflex is evident [Fig. 2(c)]. The inflammatory response was observed daily under a slit lamp from day 0 to day 18 post-immunization. EAU rats in the PBS group showed significant uveal inflammatory signs starting on day 7, which were characterized by congestion and dilatation of the iris vessels. In the PBS group, the disease was most severe on day 12 post-immunization, as evidenced by dilatation of iris vessels, anterior chamber cells and flare, obscured pupil, and severe anterior hypopyon. From 12 to 18 days post-immunization, the inflammation gradually decreased and resolved, as evidenced by the gradual absorption of anterior hypopyon, reduction of iris vascular congestion, and restored clarity of the aqueous humor [Fig. 2(d)].

Both treatment groups (DSP and SKD MCs) showed lower inflammation scores than the PBS group from the time point of subconjunctival injection on day 7 post-immunization. In the DSP group day 12 post-immunization inflammation was not well controlled, with severe hypopyon in the anterior chamber, a dim red reflex, and dilated iris vascular congestion, while the SKD MCs treatment group showed better suppression of inflammation at this time point. The anterior segment of the SKD MCs group returned to near normal on day 18 post-immunization, while the anterior chamber of the DSP group retained a small amount of unresolved anterior hypopyon and dilated iris vessels [Fig. 2(d)].

**FIG. 3.**
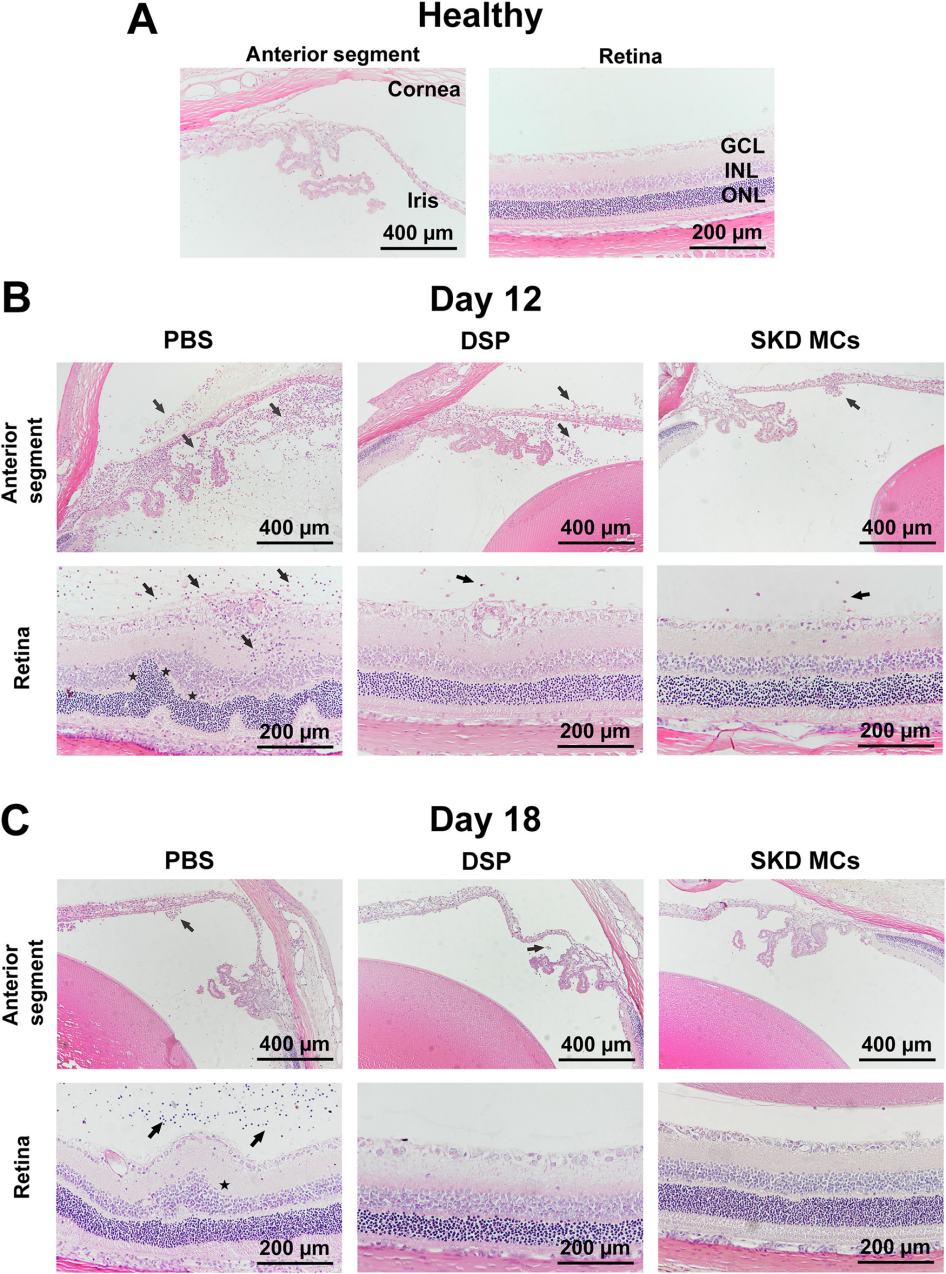
Effect of different treatments at different times on histopathologic changes in rats. Healthy rats without any intervention were used as controls (a). Histological images of the anterior segment and retina of each group of rats at day 12 (b) and day 18 (c) post-immunization, and corresponding histological images of healthy rats (a), with black arrows representing inflammatory cells and black stars representing distorted retinal structures. GCL = ganglion cell layer, INL = inner nuclear layer, ONL = outer nuclear layer.

Both eyeballs of the rats in each group were enucleated for histopathological examination at days 12 and 18 post-immunization [Figs. 3(a)–3(c)]. On day 12, both the anterior segment and the retina of the PBS group showed severe inflammatory cell exudation and infiltration. Furthermore, narrowing of the anterior chamber angle (the angle formed by the iris and the cornea) was seen in the anterior segment, and disruption of the normal structures of the retinal layers and dilatation of the retinal vessels were seen in the retina. In the DSP group, significant inflammatory cell exudation and infiltration were seen in the anterior segment, with moderate inflammatory cell infiltration. In the posterior segment, retinal vessels were slightly dilated. In the SKD MCs group, only mild inflammatory cell exudation and infiltration was apparent in the anterior segment and retina. The SKD MCs and DSP groups showed significantly less inflammation in both anterior and posterior segments than the PBS group, and the SKD MCs group showed a milder inflammatory response in the anterior segment than the DSP group. Retinal findings were similar in the two treatment groups SKD MCs and DSP, both showing that the development of inflammation was controlled [Fig. 3(b)]. On day 18, the inflammation in the PBS group had not completely subsided, inflammatory cells were still present in the anterior segment, and some retinal structures were distorted. Inflammation in the anterior segment of the SKD MCs group had subsided, with no visible inflammatory cells, while a small number of inflammatory cells remained in the anterior segment of the DSP group. Retinal pathological manifestations in the SKD MCs and DSP groups were close to normal [Fig. 3(c)].

### B. SKD MC reduces the activation and proliferation of microglia in the retina

**FIG. 4.**
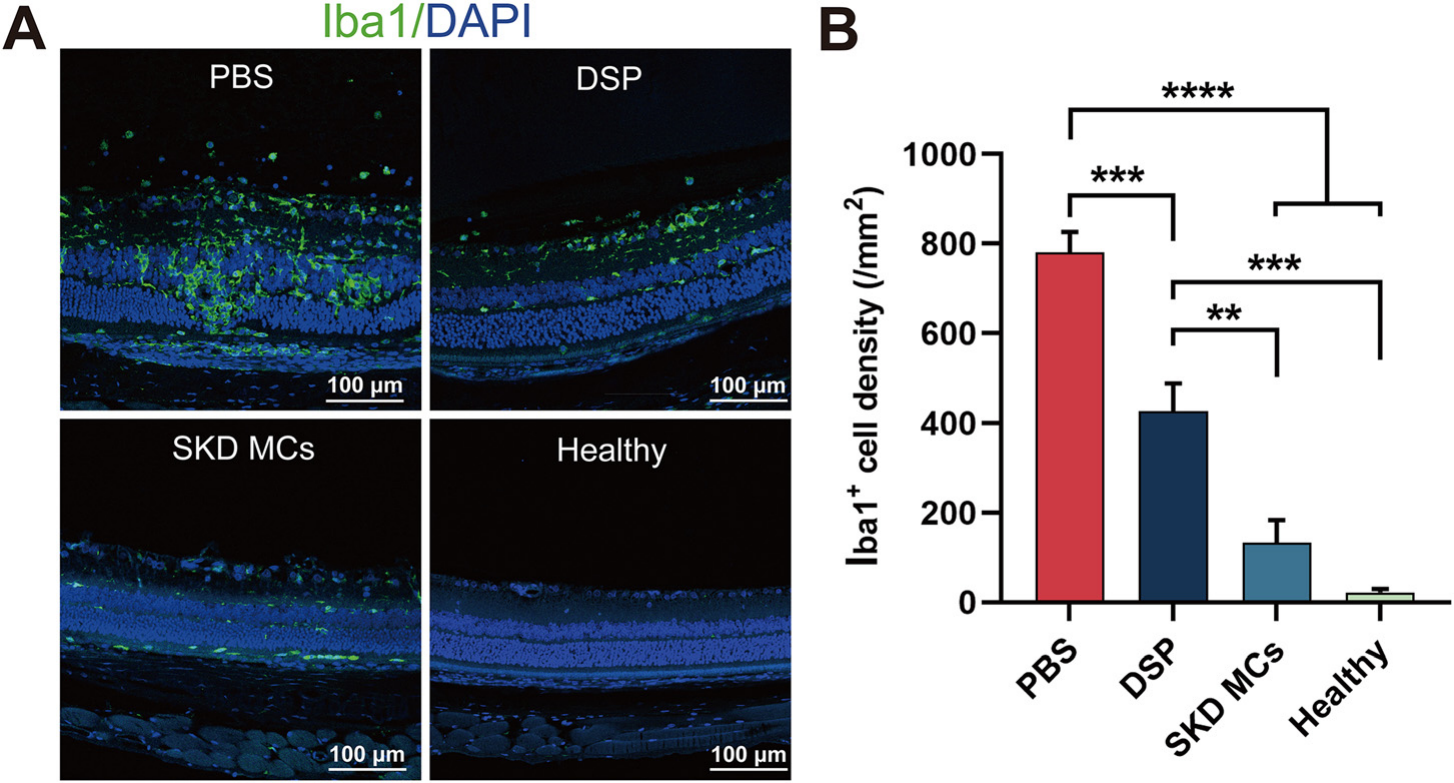
Activation of retinal microglia under different treatments. (a) Effect of different treatment regimens on positive staining of retinal Iba1. The cell nuclei were stained using DAPI (blue) and the activated microglia in the retina were stained using Iba1 (green). (b) Retinal Iba1-positive cell density under different treatment regimens. *P <* *0.05, **P <* *0.01, ***P <* *0.001, and ****P <* *0.0001. Mean ± SEM, n = 4, one-way ANOVA test.

To further explore the protective role of SKD MCs on the retina in EAU in rats, we performed immunofluorescence staining by antibodies against the microglia marker Iba1 and characterized the activation and proliferation status of microglia in the retina under different treatment regimens at day 18 post-immunization. The results showed that both SKD MCs and DSP treatment significantly reduced the expression of Iba1-positive cells in all layers of the retina [Fig. 4(a)]. In the PBS group, numerous Iba1-positive cells were stained in all retinal layers, the normal retinal structure was disrupted, and Iba1-positive cells tended to migrate to the areas of disruption. The SKD MCs group showed a significant decrease in Iba1-positive cells compared to the DSP group [Fig. 4(b)].

### C. Expression of mRNA and protein of various inflammatory cytokines

**FIG. 5.**
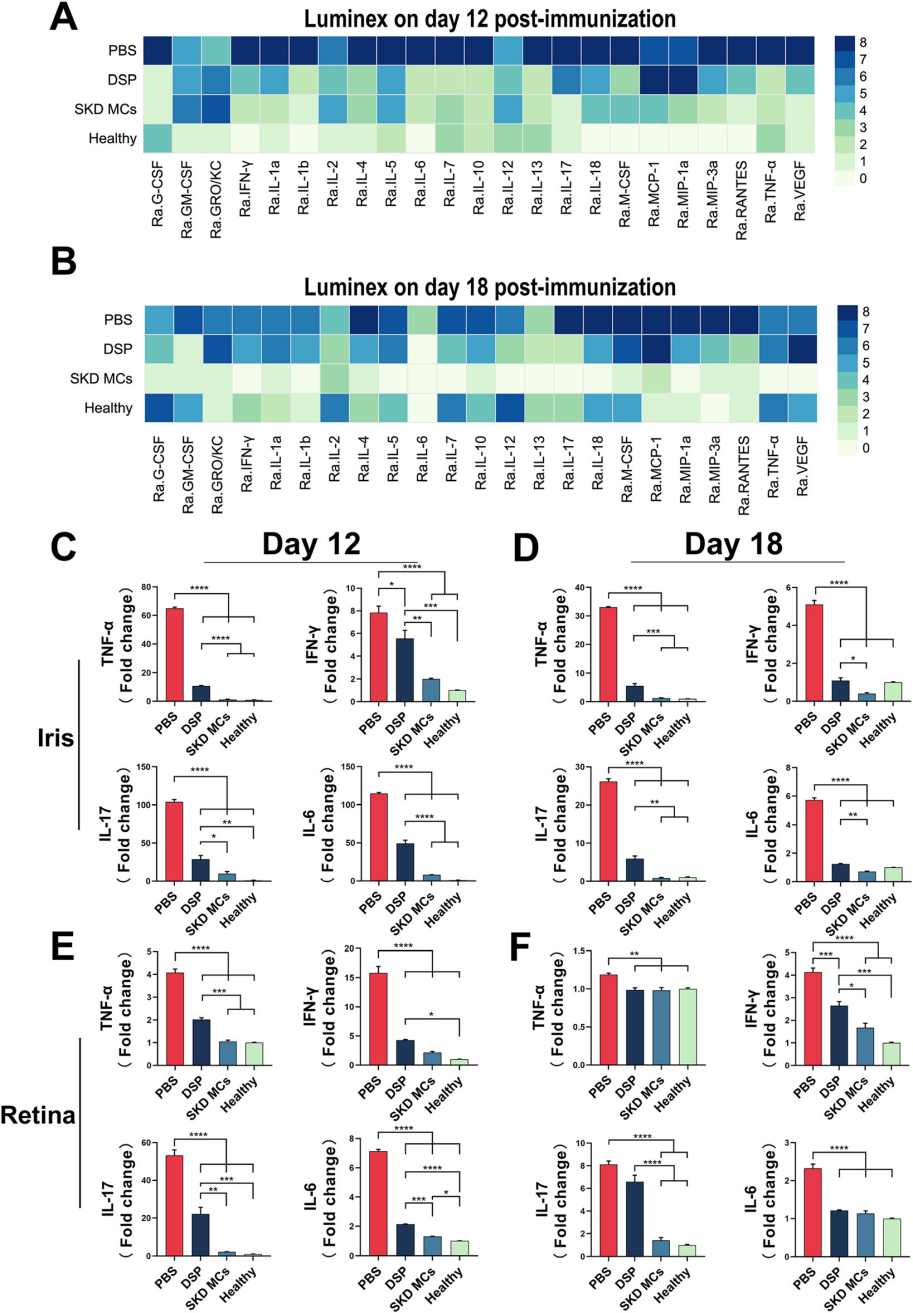
Protein and mRNA expression of inflammatory cytokines in different groups. Protein expression levels of 23 cytokines and chemokines detected by Luminex on day 12 post-immunization (a) and day 18 post-immunization (b). Expression of four inflammatory cytokine mRNAs in rat iris (c) and (d) and retinal (e) and (f) tissues on days 12 (c) and (e) and 18 (d) and (f) post-immunization. *P <* *0.05, **P <* *0.01, ***P <* *0.001, and ****P <* *0.0001. Mean ± SEM, n = 3, one-way ANOVA test.

To investigate the expression of inflammatory factors in EAU rats under different treatments, Luminex was adopted to compare the expression of 23 inflammatory cytokines and chemokines in aqueous humor between PBS, DSP, and SKD MCs treatments at the protein level. All data were normalized and plotted as heat maps, which showed that on day 12 post-immunization both DSP and SKD MCs treatments, compared to the PBS group, reduced the expression of 19 inflammatory cytokines and chemokine proteins, except granulocyte-macrophage colony-stimulating factor (GM-CSF), growth-related oncogene/keratinocyte chemokine (GRO/KC), monocyte chemoattractant protein (MCP)-1, and macrophage inflammatory protein (MIP)-1a [Fig. 5(a)]. At day 18 post-immunization, SKD MCs suppressed the expression of inflammatory cytokines more effectively than PBS and DSP groups, with protein concentrations close to or lower than those in the healthy rat group [Fig. 5(b)]. Notably, vascular endothelial growth factor (VEGF), which is closely associated with many ocular diseases, was strongly suppressed by SKD MCs (at both days 12 and 18 post-immunization).

Quantitative polymerase chain reaction (qPCR) was used to look for differences in mRNA expression of four inflammatory cytokines closely associated with EAU in the retina and iris, including tumor necrosis factor (TNF)-α, interferon (IFN)-γ, interleukin (IL)-17, and IL-6. Overall, SKD MCs significantly reduced the mRNA expression of these four cytokines [Figs. 5(c)–5(f)]. Differences varied between the two time points: at day 12 post-immunization, cytokine mRNA in the iris and retina was not statistically different between the SKD MCs and healthy groups, except for IL-6 in the retina. Expression of the four cytokine mRNA was significantly reduced in both treatments (SKD MCs and DSP) in the iris and retina compared to the PBS group. Moreover, in SKD MCs the expression of the four genes was significantly more inhibited than in the DSP group, except for IFN-γ in the retina. At day 18 post-immunization, in the retina and iris, the expression of the four genes in the SKD MCs group was not statistically different from that of the healthy group, and the expression of the four genes in the SKD MCs group was significantly lower than that in the DSP group, except for TNF-α and IL-6 in the retina [Figs. 5(d) and 5(f)]. At the anatomical level, the expression of the four genes in the SKD MCs group was significantly lower than in the DSP group in the iris tissue representing the anterior segment, at both days 12 and 18 after immunization [Figs. 5(c) and 5(d)].

### D. EAU rats treated with SKD MCs significantly preserve retinal function

**FIG. 6.**
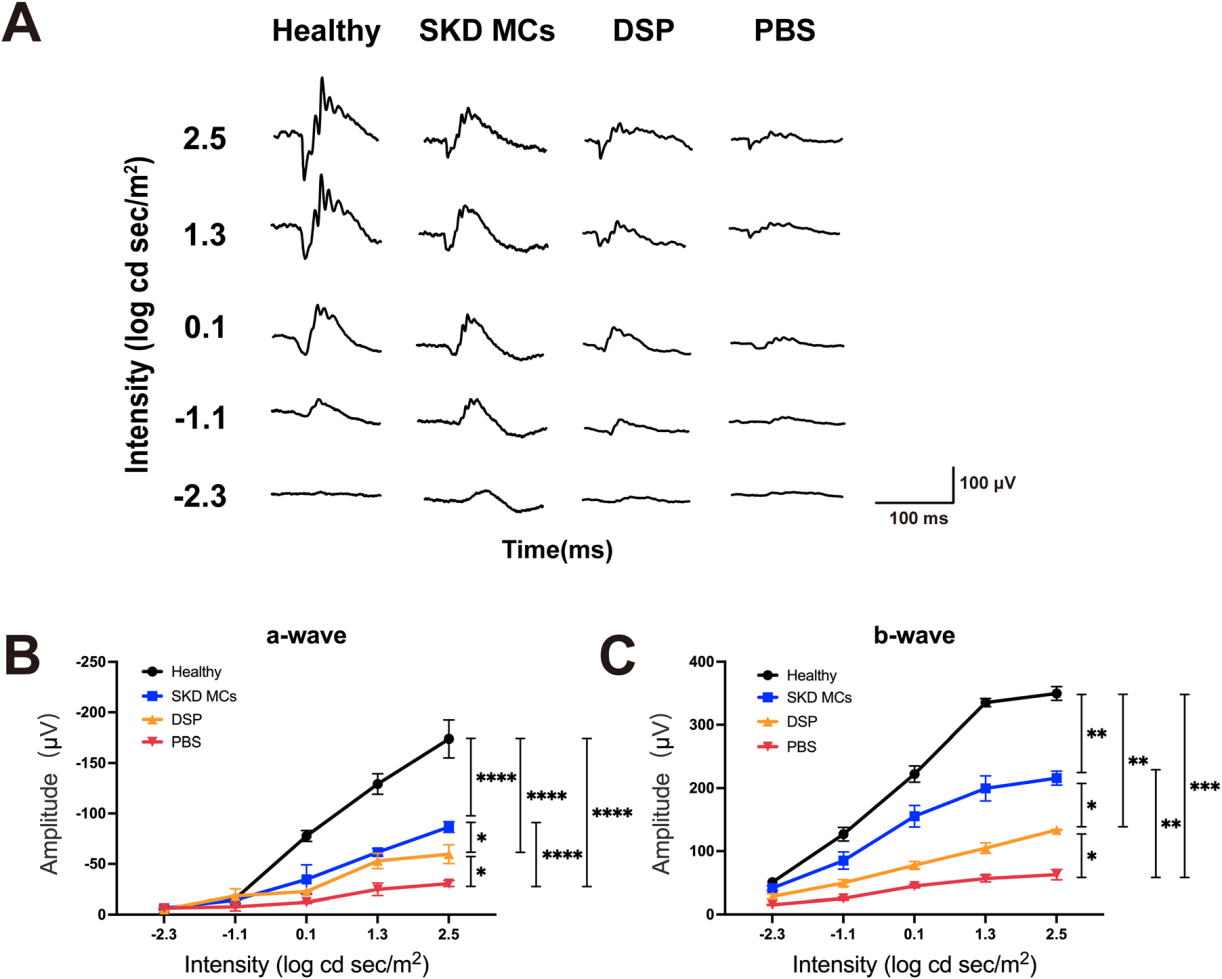
Retinal function preservation in rats under different treatments. (a) Electroretinogram responses to stimuli at five intensity levels from four groups of rats. Relationship between flash stimulus intensity and a-wave mean amplitude (b) and b-wave mean amplitude (c). Asterisks represent significance of the difference at 2.5 log cd sec/m^2^. *P <* *0.05, **P <* *0.01, ***P <* *0.001, ****P <* *0.0001. Mean ± SEM, n = 4 eyes/group, two-way ANOVA test.

On day 20 post-immunization, electroretinograms (ERGs) were recorded under dark adaptation in each group of rats to verify the preservation of retinal function by the two different treatments. ERG morphology in the SKD MCs group most closely resembled that of the healthy rats [Fig. 6(a)]. The PBS group showed lower ERG a- and b-wave amplitudes compared to the DSP, SKD MCs and healthy rat groups (*P *<* *0.05). Notably, a- and b-wave amplitudes differed significantly between the SKD MCs and DSP treatment groups, the amplitude under any light intensity stimulation being significantly higher in the SKD MCs group than in the DSP group (*P *<* *0.05) [Figs. 6(b) and 6(c)].

### E. SKD MCs treatment did not increase intraocular pressure (IOP) in rats

No statistically significant difference in intraocular pressure (IOP) was found between the SKD MCs, DSP and PBS groups during the 18 days of observation (*P* > 0.05) (supplementary material Table S3).

## III. DISCUSSION

Corticosteroids have been used for many years and are the first line medical treatments for NIU.^15^ While they are essential for rapid control of inflammation in the early treatment of uveitis, their long-term use increases the risk of adverse events, from cataract and glaucoma caused by topical treatment to life-threatening side effects such as adrenal insufficiency and Cushing-like appearance caused by systemic therapy. Moreover, NIU often requires long-term medication, and repeated dosing can reduce patient compliance and affect the effectiveness of treatment. These factors are key to treatment success, with effective control of inflammation and manageable side effects being the desired outcome.

Corticosteroids can be delivered by topical, periocular, intraocular, or systemic administration. Subconjunctival injections offer advantages over other routes of administration. They do not penetrate the eye, avoiding risks associated with intraocular injections such as endophthalmitis, rhegmatogenous retinal detachment, acute rise of IOP, and ocular hemorrhage. They are injections into the subconjunctival physiological space, where the drug penetrates through the sclera rather than the cornea, leaving a higher concentration of drug in the anterior and even posterior segments of the eye.^11,16–18^ The less invasive route may be more acceptable to patients than intravitreal injections. However, conventional drugs require repeated and multiple injections to maintain effective drug concentration levels due to short duration of action and the potential for recurrent disease episodes. Thus, a drug delivery system that allows for subconjunctival injection and sustained release of corticosteroids is expected to improve compliance and treatment outcomes for NIU patients.

We previously reported a method for the modular construction of pH-sensitive ketal-linked prodrugs of dexamethasone and demonstrated that this type of prodrug can be hydrolyzed to release dexamethasone at the site of inflammation.^19^ As part of that method, SKD, one of the ketal-linked prodrugs of dexamethasone, was prepared as MCs of different sizes (3.1 and 1.1 *μ*m). In a rat arthritis model, a single administration of the SKD MCs at the joint site achieved one month of inflammation suppression.^20^ Considering the acidic pH at many sites of inflammation, we suggest that SKD MCs have significant potential for application in other chronic inflammatory diseases.

As with the treatment of arthritis, the choice of animal model is important when assessing the feasibility of SKD in the treatment of ocular inflammation. The common animal models of uveitis to date are the endotoxin-induced model and the antigen-immune-induced EAU model, the former mainly presenting as acute anterior uveitis with a short duration of inflammation. While EAU is the classical model of human uveitis in animals, it has many similarities with human uveitis in terms of clinical and pathological manifestations, providing an effective research tool to study the pharmacological treatment of uveitis. In this study, we used interphotoreceptor retinoid binding protein (IRBP)_1177–1191_ subcutaneous immunization to induce an EAU model and obtained more severe disease manifestations and earlier onset due to the simultaneous intraperitoneal injection of 400 ng of pertussis toxin at the time of immunization induction.

In the present study, both treatment regimens significantly reduced inflammation as shown by disease scores, slit lamp anterior segment images, pathological histological images, and results from Luminex and qPCR. While SKD MCs and DSP both showed significant anti-inflammatory effects in the anterior segment of the eye, SKD MCs were more effective and longer acting, and showed a significant reduction in inflammatory scores in the middle and late stages of the disease. At these stages, we took fundus images of each group of rats to assess the effects of the different treatments on the retina. The results showed insignificant differences in image appearance between the two treatment regimens and the PBS group, probably due to the high doses of inactivated pertussis toxin and inactivated *Mycobacterium tuberculosis* used in our animal model, which made EAU more severe compared to other studies and required higher doses of steroid hormone treatment.

This study has verified the effects of different treatments on posterior segment inflammation in the posterior segment of the eye using hematoxylin and eosin (H&E) staining of retinal tissue and qPCR. The results showed that both SKD MCs and DSP treatment regimens significantly reduced inflammatory cell infiltration on pathological H&E staining results, with no significant inflammatory cell infiltration at day 18 post-immunization, and no significant difference between the two treatments. Under more sensitive qPCR detection, mRNA expression of four inflammatory cytokines including TNF-α, IFN-γ, IL-17, and IL-6 was significantly suppressed in both treatment regimens, and notably, suppression was greater in the SKD MCs group than in the DSP group.

It is well known that corticosteroids can effectively inhibit the expression and action of pro-inflammatory cytokines and induce T-cell apoptosis.^21,22^ Uveitis is currently considered to be a Th1- and Th17-mediated autoimmune disease.^23,24^ The interaction between Th1 and Th17 responses in driving uveitis in the EAU model is complex, and both are pathogenic.^25^ In order to explore the cytokine expression in EAU rats under different treatment regimens, we examined the levels of protein expression of 23 cytokines and chemokines associated with inflammation in the aqueous humor using Luminex. The Luminex results showed that SKD MCs inhibited the expression of almost all (19/23) of the cytokines and chemokines associated with inflammation, with the inhibitory effect of SKD MCs being even more significant at day 18 than at day 12 post-immunization. The Luminex results demonstrate the effectiveness of SKD MCs, and the results at the later stages of the disease (day 18 after immunization) may be the result of a prolonged and sustained release of SKD MCs. The results of Luminex were further verified using qPCR to look for differences in mRNA for TNF-α, IFN-γ, IL-17, and IL-6 in the iris and retina.

To determine the Th1 and Th17 responses, we detected the production of IFN-γ and IL-17. Subconjunctival injection of SKD MCs significantly depressed mRNA and protein expression of IFN-γ and IL-17, suggesting that the therapeutic effect of SKD MCs may be related to inhibition of Th1 or Th17 responses. IL-6 is a pleiotropic cytokine that is involved in the pathogenesis of many immune-mediated diseases, such as uveitis. Studies have shown that IL-6 plays a critical role in animal models of uveitis and that either IL-6 deficiency or anti-IL-6 treatment results in diminished uveitis and that elevated IL-6 is also detected in human uveitis.^26–29^ We found that SKD MCs treatment also significantly reduced IL-6 expression in EAU, suggesting that it may suppress the production and progression of uveitis. TNF-α is also a multifunctional cytokine and is involved in multiple signaling pathways. Many studies have observed sustained expression of TNF-α in EAU models, and increased TNF-α is also observed in retinal pigment epithelial cells and Müller cells and mediates their multiple functions,^30,31^ TNF-α also increases vascular endothelial permeability and causes tissue damage.^32,33^ In the present study, SKD MCs treatment of EAU significantly reduced intraocular TNF-α levels, which may help to diminish intraocular inflammation and tissue damage. Notably, SKD MCs treatment was also significantly better than DSP treatment in inhibiting protein expression of VEGF, suggesting that the SKD MC used here might be applied to many other ocular diseases.

We found that SKD MCs significantly inhibited the expression of cytokines, which indicates its anti-inflammatory effect, and this was corroborated by clinical findings, histopathological manifestations, preocular segmental images, and fundus images. However, some clinical patients with uveitis show no improvement in visual acuity despite normal fundus appearance after healing, perhaps due to the disruption of retinal function by inflammation.^34^ In order to verify the role of SKD MCs in protecting the visual function of rats, we examined the Ganzfeld ERG in different groups of rats before they were sacrificed on day 20 post-immunization. Studies have concluded that the ERG in rats reflects function of retinal bipolar and photoreceptor cells,^35–37^ but the b-wave is difficult to capture and record when cell damage exceeds 80% and reliability varies depending on recording and stimulation modalities.^37^ The Micron Ganzfeld ERG full field electroretinograms use Maxwellian View Illumination, in contrast to traditional large format illumination methods, and studies have shown that Ganzfeld full field stimulation provides better reproducibility and reliability.^38^ During the inflammatory phase of the disease in rats, the anterior segment response is severe, and factors such as pupillary adhesions and unclear optical media may lead to less reliable results, so we chose the remission phase of the disease (day 20 post-immunization) as the time point for testing. The ERG morphology, a-wave and b-wave amplitudes all indicated significant preservation of retinal function in the EAU of SKD MCs-treated rats compared to DSP and PBS groups. The superiority of SKD MCs in protecting retinal function may be dependent on their extremely long drug half-life.

Iba1 is a reliable marker of microglia and is expressed in increased amounts upon activation.^39^ Microglia are immune cells of the central nervous system, including the retina, and are important for the maintenance of homeostasis in the neuroretinal microenvironment.^40^ Microglia are activated during various retinal diseases, including autoimmune uveitis.^41^ Activated microglia produce inflammatory cytokines such as IL-1β and TNFα, which are important contributors to retinal photoreceptor damage, and studies have been conducted with the aim of reducing retinal damage by inhibiting microglial activation.^42–44^ In this study, the effect of different treatment modalities on retinal Iba1 expression was examined using immunofluorescence to investigate retinal damage caused by uveitis under different treatments. The results showed that Iba1 expression was significantly reduced under SKD MCs treatment, indicating that only a small number of microglia were activated in the EAU retina under SKD MCs treatment, and this situation may have preserved better retinal function.

Prolonged topical steroid hormone use is associated with an increase in IOP,^45^ but we did not observe an increase in IOP with either treatment regimen during the course of EAU, probably due to the low frequency of injection, the low dose of the drug and the short observation period.

In this study, we chose to start treatment on day 7 post-immunization, when the inflammation was in its initial stages. Inflammation persisted for about three weeks and was suppressed by SKD MCs throughout the disease phase. In our previous study, the *in vitro* hydrolysis half-lives (_t1/2_) of SKD MCs of size 3.1 *μ*M were 581 days (pH 7.4) and 200 days (pH 6.0). These long half-lives suggest that SKD MCs may have promising applications in chronic recurrent uveitis, which is a direction for our future research.

## IV. CONCLUSION

Steroids have become a common treatment for uveitis. In this study, microcrystals made from SKD administered via a single subconjunctival injection were effective in treating EAU in rats compared to DSP treatment, with significantly lower disease scores, reduced mRNA and protein expression of inflammatory cytokines, less retinal damage, reduced inflammatory cell infiltration, and more effective preservation of retinal function. SKD MCs with its very long half-life offers a promising treatment option for chronic NIU and may improve treatment outcomes through increased efficacy and improved patient compliance. Our studies indicate that SKD MCs hold promise for application in other ocular inflammatory diseases, and given that all hydrolysis by-products of SKD MCs are nontoxic and biocompatible, SKD MCs may boost the translation of anti-inflammation nanomedicines into clinical use.

## V. METHODS

### A. Preparation of SKD MCs

SKD synthesis^19^ and 3.1 *μ*m SKD MC preparation were in accordance with published methods.^20^ The SKD MC injected suspension (5 mg/ml eq. dexamethasone) was prepared by dispersing SKD MCs in 6.7 mM PBS containing 0.5% polysorbate 80 (w/w).

### B. Induction of EAU in Lewis rats and treatment protocols

A total of 46 female Lewis rats purchased from Vital River (Beijing, China) at 6–8 weeks were housed in the specific pathogen-free environment. After 1 week of acclimatization, all rats were anesthetized with 1% sodium pentobarbital (80 mg/kg) by intraperitoneal injection, then rats were immunized by subcutaneous injection of 200 *μ*l of IRBP_1177–1191_ peptide fragment (R16, ADGSSWEGVGVVPDV) (500 *μ*g/ml) into both thighs (50 *μ*l) and the base of the tail (100 *μ*l). Prior to this, IRBP was emulsified in complete Freund's adjuvant (1:1 v/v) containing 5 mg/ml of inactivated *Mycobacterium tuberculosis*, while 400 ng of pertussis toxin was administered intraperitoneally to each rat as supplementary adjuvant. The study protocol was approved by the Tianjin Nankai Hospital Animal Ethical and Welfare Committee (No. NKYY-DWLL-2021–043).

To investigate the therapeutic effect of the drug, a single group-specific subconjunctival injection was administered to rats on day 7 post-immunization. Treatment by group was as follows: (group 1) 40 *μ*l of SKD MCs (5 mg/ml eq. dexamethasone), n = 12 rats; (group 2) 40 *μ*l of DSP (5 mg/ml DSP), n = 12 rats; and (group 3) 40 *μ*l of 6.7 mM PBS containing 0.5% polysorbate 80(w/w), n = 12 rats. After injection, to protect the cornea, erythromycin eye ointment was applied to prevent drying. In addition, 10 healthy rats without intervention were used as a healthy control group. Four rats in each group were sacrificed on day 12 and 18 post-immunization and samples were taken for testing.

### C. Clinical observations and inflammation scores

Using a slit lamp, the researchers looked for signs of inflammatory changes in the ocular anterior segment daily starting on day 0 post-immunization, and their severity was scored and recorded according to established scoring criteria until all experimental rats were sacrificed on day 18 post-immunization.^46^ On day 18 post-immunization, rats' pupils were dilated with 0.5% tropicamide and fundus images were recorded using the Micron IV retinal imaging microscope (Phoenix Research Labs, Pleasanton, CA, USA).

### D. Histology

On day 12 and 18 post-immunization, the rats were sacrificed and the eyeballs were harvested, fixed in 4% paraformaldehyde for three days, and then embedded in paraffin. Sections of 4 *μ*m thickness were stained with standard H&E. Stained sections were evaluated under a light microscope (Olympus CX41, Tokyo, Japan) to grade the severity of inflammation.

### E. Immunofluorescence (IF)

On day 18 post-immunization, rat eyeballs were cut into 5 *μ*m slices for immunofluorescence detection after embedding in paraffin. Briefly, the slides were dewaxed, antigenically repaired with ethylenediaminetetraacetic acid citrate (pH 6.0) antigen repair buffer (G1202, Servicebio, Wuhan, China), blocked with 10% goat serum for one hour, and incubated overnight in diluted primary antibody (anti-rabbit Iba-1, A19776, 1:300, ABclonal, Wuhan, China). Secondary antibodies (goat anti-rabbit Alexa Fluor 488, AS053, 1:300, ABclonal, Wuhan, China) were then incubated for 60 min at room temperature and protected from light, and slides were washed three times, followed by 4′,6-diamidino-2-phenylindole nuclear staining (G1012, Servicebio, Wuhan, China). Sections were washed again and sealed with anti-fade blocker (G1401, Servicebio, Wuhan, China) and analyzed using laser scanning confocal microscopy (Leica, Wetzlar, Germany). Cell count was conducted using ImageJ software (National Institutes of Health, Bethesda, MD, USA).

### F. Luminex analysis

The following cytokines and chemokines were detected using the Bio-Plex Pro Rat 23-plex kit (Bio-Rad, CA, USA): IL-1a, IL-1b, IL-2, IL-4, IL-5, IL-6, IL-7, IL-10, IL-12, IL-13, IL-17, IL-18, granulocyte colony-stimulating factor (G-CSF), GM-CSF, GRO\KC, IFN-γ, macrophage colony-stimulating factor (M-CSF), MCP-1, MIP-1a, MIP-3a, RANTES, TNF-α, and VEGF. Briefly, 25 *μ*l of rat aqueous humor was collected at day 18 post-immunization in 96-well plates that had been embedded with microbeads and incubated for one hour, then incubated with the detection antibody for 30 min. After several washes, Streptavidin-PE was added to each well and incubated for 10 min, after which cytokine and chemokine expression was measured using the Luminex 200 System (Luminex Corporation, Austin, TX, USA).

### G. Extraction of total RNA and qPCR

The mRNA expression levels of four inflammatory cytokines TNF-α, IFN-γ, IL-17, and IL-6 were measured using qPCR. Total RNA was extracted from the iris and retina of rats in each group using the Universal RNA Purification Kit (EZBioscience, Roseville, USA) on day 12 and day 18 post-immunization, following the instructions provided by the manufacturer. The concentration of total RNA was determined using NanoDrop 8000 (Thermo Fisher Scientific, Waltham, MA, USA). RNA was reverse transcribed into cDNA using the All-in-one Reverse Transcription Kit (EZBioscience, Roseville, USA). Quantitative PCR analysis was conducted using the LightCycler® 96 System (Roche, Mannheim, Germany) and SYBR Green qPCR Master Mix (EZBioscience, Roseville, USA), and glyceraldehyde-3-phosphate dehydrogenase was used for data normalization. Data were analyzed using the 2^-ΔΔCT^ method and all primers were purchased from Sangon Biotech (Shanghai, China).

### H. Electroretinogram

ERGs were recorded at day 20 post-immunization and after dark adaptation overnight, according to the manufacturer's instructions (Phoenix Research Labs, Pleasanton, CA, USA). Briefly, after deeply anesthetizing the rats, their pupils were dilated with 5% tropicamide. The rats were fixed in the prone position, and each electrode was placed according to the instrument instructions. An electrode was placed in contact with the cornea after applying 2.5% Hypromellose (OCuSoft, Rosenberg, TX, USA), the reference electrode was placed subcutaneously between the ears, and the ground electrode was placed subcutaneously under the tail. ERGs were recorded at stimulus intensities of −2.3, −1.1, 0.1, 1.3, and 2.5 log cd sec/m^2^, respectively.

### I. Recording of IOP

On day 0, day 12, and day 18 post-immunization, before the rats were sacrificed, IOP measurements were made using a rodent-specific tonometer (Icare, Helsinki, Finland) according to the manufacturer's instructions, and the IOP recorded in each eye was averaged over three measurements.

### J. Statistical analysis

All data were analyzed using GraphPad Prism 8 software (GraphPad Software, CA, USA). The experimental results are shown as the mean of at least three replicates. Statistical comparisons were made using one-way ANOVA with Tukey's test for multiple comparisons and two-way ANOVA with Sidak test for multiple comparisons. Differences were considered significant at P values less than 0.05.

## SUPPLEMENTARY MATERIAL

See the supplementary material for the appearance of SKD MCs under the conjunctiva. Tables show EAU disease score, target gene primers, and IOP values.

## Data Availability

The data that support the findings of this study are available within the article and its supplementary material.

## References

[1] E. Miserocchi *et al.*, “ Review on the worldwide epidemiology of uveitis,” Eur. J. Ophthalmol. 23, 705 (2013). 10.5301/ejo.5000278 23661536

[2] M. D. de Smet *et al.*, “ Understanding uveitis: The impact of research on visual outcomes,” Prog. Retinal Eye Res. 30, 452 (2011). 10.1016/j.preteyeres.2011.06.005 21807112

[3] M. S. A. Suttorp-Schulten and A. Rothova , “ The possible impact of uveitis in blindness: A literature survey,” Br. J. Ophthalmol. 80, 844 (1996). 10.1136/bjo.80.9.844 8962842 PMC505625

[4] J. Pan , M. Kapur , and R. McCallum , “ Noninfectious immune-mediated uveitis and ocular inflammation,” Curr. Allergy Asthma Rep. 14, 409 (2014). 10.1007/s11882-013-0409-1 24338488

[5] L. M. Valdes and L. Sobrin , “ Uveitis therapy: The corticosteroid options,” Drugs 80, 765 (2020). 10.1007/s40265-020-01314-y 32350761

[6] R. Gaudana *et al.*, “ Ocular drug delivery,” AAPS J. 12, 348 (2010). 10.1208/s12248-010-9183-3 20437123 PMC2895432

[7] A. K. McDonough , J. R. Curtis , and K. G. Saag , “ The epidemiology of glucocorticoid-associated adverse events,” Curr. Opin. Rheumatol. 20, 131 (2008). 10.1097/BOR.0b013e3282f51031 18349741

[8] J. N. Hoes *et al.*, “ Adverse events of low- to medium-dose oral glucocorticoids in inflammatory diseases: A meta-analysis,” Ann. Rheum. Dis. 68, 1833 (2009). 10.1136/ard.2008.100008 19066177

[9] J. Zarranz-Ventura *et al.*, “ Multicenter study of intravitreal dexamethasone implant in noninfectious uveitis: Indications, outcomes, and reinjection frequency,” Am. J. Ophthalmol. 158, 1136 (2014). 10.1016/j.ajo.2014.09.003 25217856

[10] O. Weijtens *et al.*, “ High concentration of dexamethasone in aqueous and vitreous after subconjunctival injection,” Am. J. Ophthalmol. 128, 192 (1999). 10.1016/S0002-9394(99)00129-4 10458175

[11] M. R. Jain and S. Srivastava , “ Ocular penetration of hydrocortisone and dexamethasone into the aqueous humour after subconjunctival injection,” Trans. Ophthalmol. Soc. U.K. 98, 63–65 (1978). 373174

[12] L. Huang *et al.*, “ Engineering of small-molecule lipidic prodrugs as novel nanomedicines for enhanced drug delivery,” J. Nanobiotechnol. 20, 49 (2022). 10.1186/s12951-022-01257-4 PMC8785568 35073914

[13] J. Rautio *et al.*, “ The expanding role of prodrugs in contemporary drug design and development,” Nat. Rev. Drug Discovery 17, 559 (2018). 10.1038/nrd.2018.46 29700501

[14] C. Alvarez-Lorenzo and A. Concheiro , “ Smart drug delivery systems: From fundamentals to the clinic,” Chem. Commun. 50, 7743 (2014). 10.1039/C4CC01429D 24805962

[15] S. A. Gaballa *et al.*, “ Corticosteroids in ophthalmology: Drug delivery innovations, pharmacology, clinical applications, and future perspectives,” Drug Delivery Transl. Res. 11, 866 (2021). 10.1007/s13346-020-00843-z 32901367

[16] M. A. Awan *et al.*, “ Penetration of topical and subconjunctival corticosteroids into human aqueous humour and its therapeutic significance,” Br. J. Ophthalmol. 93, 708 (2009). 10.1136/bjo.2008.154906 19293163

[17] F. Rafiei , H. Tabesh , and F. Farzad , “ Sustained subconjunctival drug delivery systems: Current trends and future perspectives,” Int. Ophthalmol. 40, 2385 (2020). 10.1007/s10792-020-01391-8 32383131

[18] J. Fu *et al.*, “ Subconjunctival delivery of dorzolamide-loaded poly(ether-anhydride) microparticles produces sustained lowering of intraocular pressure in rabbits,” Mol. Pharm. 13, 2987 (2016). 10.1021/acs.molpharmaceut.6b00343 27336794 PMC5088785

[19] Y. Xu *et al.*, “ Modular acid-activatable acetone-based ketal-linked nanomedicine by dexamethasone prodrugs for enhanced anti-rheumatoid arthritis with low side effects,” Nano Lett. 20, 2558 (2020). 10.1021/acs.nanolett.9b05340 32167768

[20] Y. Xu *et al.*, “ Intra-articular injection of acid-sensitive stearoxyl-ketal-dexamethasone microcrystals for long-acting arthritis therapy,” Asian J. Pharm. Sci. 16, 213 (2020). 10.1016/j.ajps.2020.07.002 33995615 PMC8105419

[21] K. Ito , K. F. Chung , and I. M. Adcock , “ Update on glucocorticoid action and resistance,” J. Allergy Clin. Immunol. 117, 522 (2006). 10.1016/j.jaci.2006.01.032 16522450

[22] T. Rhen and J. A. Cidlowski , “ Antiinflammatory action of glucocorticoids—New mechanisms for old drugs,” N. Engl. J. Med. 353, 1711 (2005). 10.1056/NEJMra050541 16236742

[23] D. Wakefield and A. Lloyd , “ The role of cytokines in the pathogenesis of inflammatory eye disease,” Cytokine 4(1), 1–17 (1992). 10.1016/1043-4666(92)90028-P 1617154

[24] J. E. Weinstein and K. L. Pepple , “ Cytokines in uveitis,” Curr. Opin. Ophthalmol. 29, 267 (2018). 10.1097/ICU.0000000000000466 29521875 PMC7199509

[25] T. Yoshimura *et al.*, “ Differential roles for IFN-γ and IL-17 in experimental autoimmune uveoretinitis,” Int. Immunol. 20, 209 (2008). 10.1093/intimm/dxm135 18156624

[26] P. Lin , “ Targeting interleukin-6 for noninfectious uveitis,” Clin. Ophthalmol. 9, 1697 (2015). 10.2147/OPTH.S68595 26392750 PMC4574854

[27] J. Tode *et al.*, “ Intravitreal injection of anti-Interleukin (IL)-6 antibody attenuates experimental autoimmune uveitis in mice,” Cytokine 96, 8 (2017). 10.1016/j.cyto.2017.02.023 28267649

[28] T. Yoshimura *et al.*, “ Involvement of Th17 cells and the effect of anti-IL-6 therapy in autoimmune uveitis,” Rheumatology 48, 347 (2009). 10.1093/rheumatology/ken489 19164426 PMC2722800

[29] W. Chen *et al.*, “ Cytokine expression profile in aqueous humor and sera of patients with acute anterior uveitis,” Curr. Mol. Med. 15, 543 (2015). 10.2174/1566524015666150731100012 26238370

[30] Y. de Kozak *et al.*, “ Tumor necrosis factor and nitric oxide production by resident retinal glial cells from rats presenting hereditary retinal degeneration,” Ocul. Immunol. Inflammation 5, 85 (1997). 10.3109/09273949709085056 9234372

[31] G. M. Holtkamp *et al.*, “ Retinal pigment epithelium-immune system interactions: Cytokine production and cytokine-induced changes,” Prog. Retinal Eye Res. 20, 29 (2001). 10.1016/S1350-9462(00)00017-3 11070367

[32] J. D. Luna *et al.*, “ Blood-retinal barrier (BRB) breakdown in experimental autoimmune uveoretinitis: Comparison with vascular endothelial growth factor, tumor necrosis factor α, and interleukin-1β-mediated breakdown,” J. Neurosci. Res. 49, 268 (1997). 10.1002/(SICI)1097-4547(19970801)49:3<268::AID-JNR2>3.0.CO;2-A 9260738

[33] X. Meng *et al.*, “ Preventive effect of chrysin on experimental autoimmune uveitis triggered by injection of human IRBP peptide 1–20 in mice,” Cell Mol. Immunol. 14, 702 (2017). 10.1038/cmi.2015.107 26996065 PMC5549600

[34] A. H. Brouwer *et al.*, “ Electroretinogram abnormalities in non-infectious uveitis often persist,” Acta Ophthalmol. 98, 627 (2020). 10.1111/aos.14401 32190989 PMC7496825

[35] B. V. Bui and B. Fortune , “ Ganglion cell contributions to the rat full-field electroretinogram,” J. Physiol. 555, 153 (2004). 10.1113/jphysiol.2003.052738 14578484 PMC1664823

[36] I. Ranchon *et al.*, “ Light-induced variations of retinal sensitivity in rats,” Curr. Eye Res. 17, 14 (1998). 10.1076/ceyr.17.1.14.5250 9472466

[37] T. Sugawara , P. A. Sieving , and R. A. Bush , “ Quantitative relationship of the scotopic and photopic ERG to photoreceptor cell loss in light damaged rats,” Exp. Eye Res. 70, 693 (2000). 10.1006/exer.2000.0842 10870528

[38] A. U. Bayer *et al.*, “ Evaluation of different recording parameters to establish a standard for flash electroretinography in rodents,” Vision Res. 41, 2173 (2001). 10.1016/S0042-6989(01)00103-1 11448710

[39] M. Schwabenland *et al.*, “ Analyzing microglial phenotypes across neuropathologies: A practical guide,” Acta Neuropathol. 142, 923 (2021). 10.1007/s00401-021-02370-8 34623511 PMC8498770

[40] S. M. Silverman and W. T. Wong , “ Microglia in the retina: Roles in development, maturity, and disease,” Annu. Rev. Vis. Sci. 4, 45 (2018). 10.1146/annurev-vision-091517-034425 29852094

[41] A. Dagkalis *et al.*, “ CX3CR1-deficiency is associated with increased severity of disease in experimental autoimmune uveitis,” Immunology 128, 25 (2009). 10.1111/j.1365-2567.2009.03046.x 19689733 PMC2747136

[42] B. Peng *et al.*, “ Suppression of microglial activation is neuroprotective in a mouse model of human retinitis pigmentosa,” J. Neurosci. 34, 8139 (2014). 10.1523/JNEUROSCI.5200-13.2014 24920619 PMC6608244

[43] Y. Okunuki *et al.*, “ Retinal microglia initiate neuroinflammation in ocular autoimmunity,” Proc. Natl. Acad. Sci. U. S. A. 116, 9989 (2019). 10.1073/pnas.1820387116 31023885 PMC6525481

[44] J. Zhou *et al.*, “ A combination of inhibiting microglia activity and remodeling gut microenvironment suppresses the development and progression of experimental autoimmune uveitis,” Biochem. Pharmacol. 180, 114108 (2020). 10.1016/j.bcp.2020.114108 32569628

[45] H. L. Cantrill *et al.*, “ Comparison of in vitro potency of corticosteroids with ability to raise intraocular pressure,” Am. J. Ophthalmol. 79, 1012 (1975). 10.1016/0002-9394(75)90687-X 1173539

[46] R. K. Agarwal and R. R. Caspi , “ Rodent models of experimental autoimmune uveitis,” Methods Mol. Med. 102, 395 (2004). 10.1385/1-59259-805-6:395 15286397

